# Boosting the diagnostic power of amyloid-β PET using a data-driven spatially informed classifier for decision support

**DOI:** 10.1186/s13195-021-00910-8

**Published:** 2021-11-10

**Authors:** Ashwin V. Venkataraman, Wenjia Bai, Alex Whittington, James F. Myers, Eugenii A. Rabiner, Anne Lingford-Hughes, Paul M. Matthews, for the Alzheimer’s Disease Neuroimaging Initiative

**Affiliations:** 1 Department of Brain Sciences, Imperial College London, 5th Floor Burlington Danes Building, 160 Du Cane Road, London, W12 0NN UK; 2 UK Dementia Research Institute at Imperial College London, London, UK; 3 Data Science Institute, Imperial College London, London, UK; 4 Invicro LLC, London, UK

**Keywords:** Alzheimer’s, Amyloid clusters, Amyloid PET, Machine learning, Clustering, Automated decision

## Abstract

**Background:**

Amyloid-β (Aβ) PET has emerged as clinically useful for more accurate diagnosis of patients with cognitive decline. Aβ deposition is a necessary cause or response to the cellular pathology of Alzheimer’s disease (AD). Usual clinical and research interpretation of amyloid PET does not fully utilise all information regarding the spatial distribution of signal. We present a data-driven, spatially informed classifier to boost the diagnostic power of amyloid PET in AD.

**Methods:**

Voxel-wise *k*-means clustering of amyloid-positive voxels was performed; clusters were mapped to brain anatomy and tested for their associations by diagnostic category and disease severity with 758 amyloid PET scans from volunteers in the AD continuum from the Alzheimer’s Disease Neuroimaging Initiative (ADNI). A machine learning approach based on this spatially constrained model using an optimised quadratic support vector machine was developed for automatic classification of scans for AD vs non-AD pathology.

**Results:**

This classifier boosted the accuracy of classification of AD scans to 81% using the amyloid PET alone with an area under the curve (AUC) of 0.91 compared to other spatial methods. This increased sensitivity to detect AD by 15% and the AUC by 9% compared to the use of a composite region of interest SUVr.

**Conclusions:**

The diagnostic classification accuracy of amyloid PET was improved using an automated data-driven spatial classifier. Our classifier highlights the importance of considering the spatial variation in Aβ PET signal for optimal interpretation of scans. The algorithm now is available to be evaluated prospectively as a tool for automated clinical decision support in research settings.

**Supplementary Information:**

The online version contains supplementary material available at 10.1186/s13195-021-00910-8.

## Background

Alzheimer’s disease (AD) is characterised by amyloid-β (Aβ) [1–3] and tau deposition [4] in the brain. This neuropathology progresses with disease symptoms and severity. AD affects 50 million people worldwide and, while it has no cure, its diagnosis has a major impact on people, their families and clinical care [5]. The number of people affected is forecast to triple by 2050 [6] making confident early diagnosis and monitoring ever more critically important.

Aβ deposition in the brain is a necessary element [7] in the development of AD [8]. Higher brain Aβ deposition is associated with faster memory decline [9] and regional hypometabolism in distally connected brain regions [10]. Amyloid PET in life correlates well with *post mortem* Aβ deposition [11]. Amyloid PET thus has emerged as clinically useful for the diagnosis of patients with cognitive decline in clinic [12, 13]. Amyloid PET has also been used both to better ensure patient diagnoses for inclusion and as an endpoint measure in clinical trials [14–16]. Supplementation of clinical assessment with amyloid PET increases the accuracy of diagnosis and change in patient management [13, 17–19]. However, one challenge is that cognitively normal individuals and other non-AD pathologies also can show Aβ deposition [20, 21] with differential regional vulnerabilities.

The neuropathology in AD is distinguished by a neuroanatomically distinct pattern of Aβ and tau deposition, with a hypothesised mechanistic relationship between amyloid deposition and tau [1, 3, 4]. Early detection of pathological amyloid deposition may help to identify people at higher risk of progression to AD. Regional vulnerabilities to the accumulation of pathological proteins and neurodegeneration may arise as a consequence of their functional anatomy. Defining the changing regional distribution of the Aβ PET signal with progression thus may help both to characterise disease stages and understand their clinical expression [22]. More recently, Tau PET has been shown to be useful in discriminating AD pathology; however, the high cost and low availability, and relative later life deposition, make it practically difficult at present [23].

Most current clinical interpretation of amyloid PET in real-world settings relies on a visual read of scans. The trained interpreter delineates cortical grey-white matter differentiation of amyloid PET signal, where reduced/absent differentiation of tracer uptake indicates Aβ positivity [24]. A metric such as a global standardised uptake value ratio (SUVr) may be included in reports, but only limited spatial distribution information is used. Alternative quantitative metrics are available, but they do not explicitly take into account relationships between neuroanatomical variations in Aβ accumulation and the progression of cognitive impairment [25]. Regional and whole-brain specific information by use of a composite cortical SUVr mask to assess global amyloid burden is the primary outcome measure in many studies [26–28].

Our end goal was to develop an automated decision support to differentiate between AD and non-AD subjects based on amyloid PET by utilising disease-relevant neuroanatomical (spatial) information. To do this we developed Aβ masks using a data-driven methodology with amyloid PET data from the Alzheimer’s Disease Neuroimaging Initiative (ADNI). The de novo Aβ cluster masks generated and clinical classifications of subjects then were used to optimise a classifier to discriminate AD vs non-AD pathology compared to commonly used alternative spatial constraints. In addition, we used the de novo Aβ clusters to further understand the behaviour of Aβ and regional vulnerabilities to disease.

## Methods

**Fig. 1:**
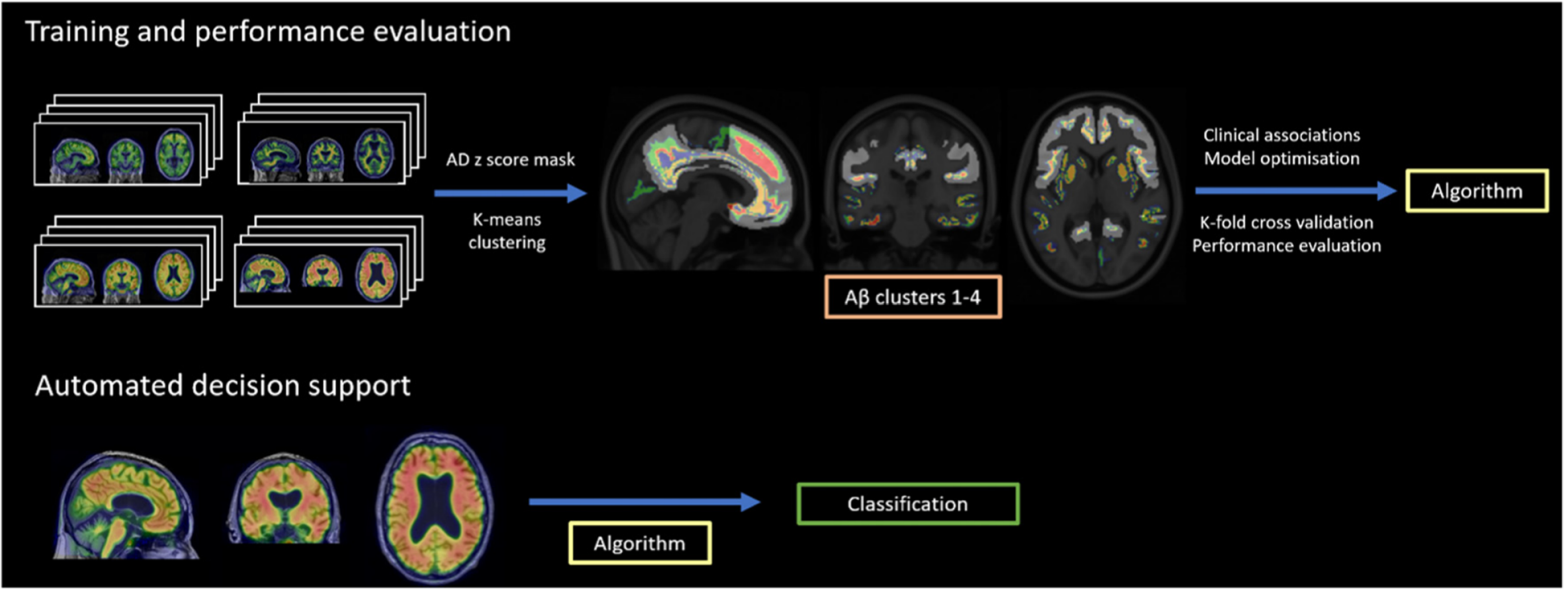
Overview of study methods: Training and performance evaluation shows the input which is a combination of [^18^F] Florbetapir PET and T1 structural MRI following co-registration, segmentation and registration to MNI 152 space stratified by group. This is used to create a binarized mask of the AD *z*-score voxels to focus where most variance of Aβ occurs in AD, and this is applied to the whole data set with *k*-means clustering to map cluster results to anatomy where Aβ clusters 1–4 are shown. Clinical associations, model testing and optimisation, and performance evaluation compared to other spatial methods are conducted to form an algorithm. This algorithm allows the input of a new [^18^F] Florbetapir PET and T1 structural MRI and classifies the scan allowing for automated decision support

Figure 1 shows a high-level overview of the methods used in the study.

### Data

Data used in the preparation of this article were obtained from the Alzheimer’s Disease Neuroimaging Initiative (ADNI) database (adni.loni.usc.edu). The ADNI was launched in 2003 as a public-private partnership, led by Principal Investigator Michael W. Weiner, MD. The primary goal of ADNI has been to test whether serial magnetic resonance imaging (MRI), positron emission tomography (PET), other biological markers, and clinical and neuropsychological assessment can be combined to measure the progression of mild cognitive impairment (MCI) and early Alzheimer’s disease (AD). ADNI received US ethical approval from 58 study locations with all participants providing informed consent (ClinicalTrials.gov identifier: NCT01231971).

### Participants

Participants from the ADNI database comprised cognitively normal (CN), early mild cognitive impairment (EMCI), late mild cognitive impairment (LMCI) and Alzheimer’s disease (AD) with available [^18^F] Florbetapir amyloid PET and corresponding T1 MPRAGE structural MRI scans (total *n* = 758), aged 55–90 years old.

Subjects were classified into CN, MCI (early and late) and AD by clinical history and neuropsychological evaluation. CN subjects had Mini-Mental State Examination (MMSE) scores of 24–30 with no memory complaints and a Clinical Dementia Rating (CDR) of zero. MCI subjects had MMSE scores of 24–30, CDR scores of 0.5 and objective memory impairment. AD patients had MMSE scores of 20–26 and CDR scores greater than 0.5. Full inclusion and exclusion criteria for ADNI are described elsewhere [29].

### Imaging data

Each subject underwent a 20-min [^18^F] Florbetapir PET scan 50 min post-injection (370±37 MBq) according to the standardised ADNI protocol. Image pre-processing steps are described elsewhere (adni.loni.usc.edu/methods/pet-analysis/pre-processing).

[^18^F]Florbetapir PET were analysed using MIAKAT^TM^ (version 4.3.7, miakat.org), that implements MATLAB (version R2019a), FSL (version 5.0.4) functions for brain extraction and SPM12 (fil.ion.ucl.ac.uk/spm) for image segmentation and registration. [^18^F]Florbetapir PET were nonlinearly registered into MNI152 space with DARTEL. Structural MRI images were segmented into GM (grey matter) and WM (white matter) with SPM12 and registered to a group average template. The group average template was then registered to MNI152 space. Each subjects’ [^18^F]Florbetapir PET SUVr image was registered to the corresponding MRI using a rigid-body registration. Individuals DARTEL flow field and template transformation was applied without modulation resulting in [^18^F]Florbetapir images in MNI152 space.

SUVr data were quantified by dividing each SUV image by its mean cerebellar GM reference. Mean and standard deviation voxel-wise GM masked images of each group (CN, EMCI, LMCI, AD) were created. Three *z*-score GM masked image of each group (EMCI, LMCI and AD) relative to CN was output where the group *z*-score = (group mean − CN mean)/CN standard deviation). A de-noised binarised mask (at SUVr threshold 1.1 [30–32]) of the AD *z-*score voxels was created to focus where most variance of Aβ occurs in AD.

### Cluster-based method

The AD *z*-score thresholded mask was applied to the whole [^18^F]Florbetapir PET images dataset (all individual unthresholded GM masked images in MNI152). Voxels from 758 subjects were clustered into *k* groups according to their SUVr intensities. To do so all spatially constrained voxels for each subject were concatenated into a long vector and a one-dimensional *k*-means clustering of a 1000 iterations per voxel using MATLAB was performed across all subjects between corresponding masked voxels for *k* = 2–13, where *k* denotes the number of clusters. For each voxel, the membership of the cluster it belongs to was determined, and the output clustering results were reshaped from a long vector into brain image space, mapping the clusters to anatomy.

In order to form clinical associations of each *k* cluster and its divisions with disease category, each group (CN, EMCI, LMCI, AD) GM masked mean image was constrained by the AD *z*-score binarised mask. For each *k* cluster output image, its divisions were created and saved as a mask (*n* = 90 masks for *k* = 2–13). Each *k* cluster division mask was applied to each group. GM masked mean image was constrained by the AD *z*-score binarised mask in order to focus on AD relevant spatial information.

### Associations with diagnostic category and clinical variables

We had 4 clinically defined groups of subjects and we considered them as defining a progression of disease in 4 stages (CN, EMCI, LMCI, and AD). We considered the representation of voxels in each cluster by clinical stage and counted the number of voxels in each group within each k cluster’s divisions. This process of applying the k division masks and counting positive voxels was also repeated for each individual subject, then grouped by diagnostic category and ordered by progression. An amyloid positive voxel was determined accordingly to commonly associated thresholds for positivity [30, 31]. Additionally, spillover from WM was assessed by calculating the mean SUVr in individual subjects within a WM mask and stratifying by group. MMSE and CDR correlations were explored for Aβ clusters 1–4 for scans from 730 subjects where an MMSE was recorded within 6 months of the [^18^F]Florbetapir PET and for 758 subject scans for which a CDR score was recorded within 3 months of [^18^F]Florbetapir PET.

### Classification and performance evaluation

In order to classify AD vs non-AD, a support vector machine (SVM) was chosen [33]. An optimum SVM was selected by adjusting kernels, and serially testing classifications using quadratic, linear, and coarse Gaussian SVMs. Twentyfold cross-validation using a quadratic SVM across 342 subjects and 2 response variables (AD, CN) was performed in MATLAB. The optimised machine learning classifier for the following inputs was assessed for each individual subject for a total of 6 features amyloid PET positive voxel clusters number 1–4 (4), GM voxels positive, GM mean SUVr). The performance of all 6 features were compared to the de novo Aβ clusters 1–4 (4 features), and features of existing measures namely composite SUVr, and 2 features of GM voxels positive, GM mean SUVr using logistic regression. Confusion matrices, accuracy, sensitivity, specificity, AUC-ROC, alongside error rates were evaluated for the 4 inputs.

## Results

### Demographics

Seven hundred fifty-eight participants with available [^18^F]Florbetapir PET scans for processing were included in the analysis. Demographic data are shown in Table 1.

**Table 1:** Clinical demographics of participants

	CN	EMCI	LMCI	AD
*n*	199	196	198	165
Age (SD)	76 (6)	70 (7)	72 (8)	76 (8)
% male	54	57	49	55

### Data-driven cross-sectional staging

**Fig. 2:**
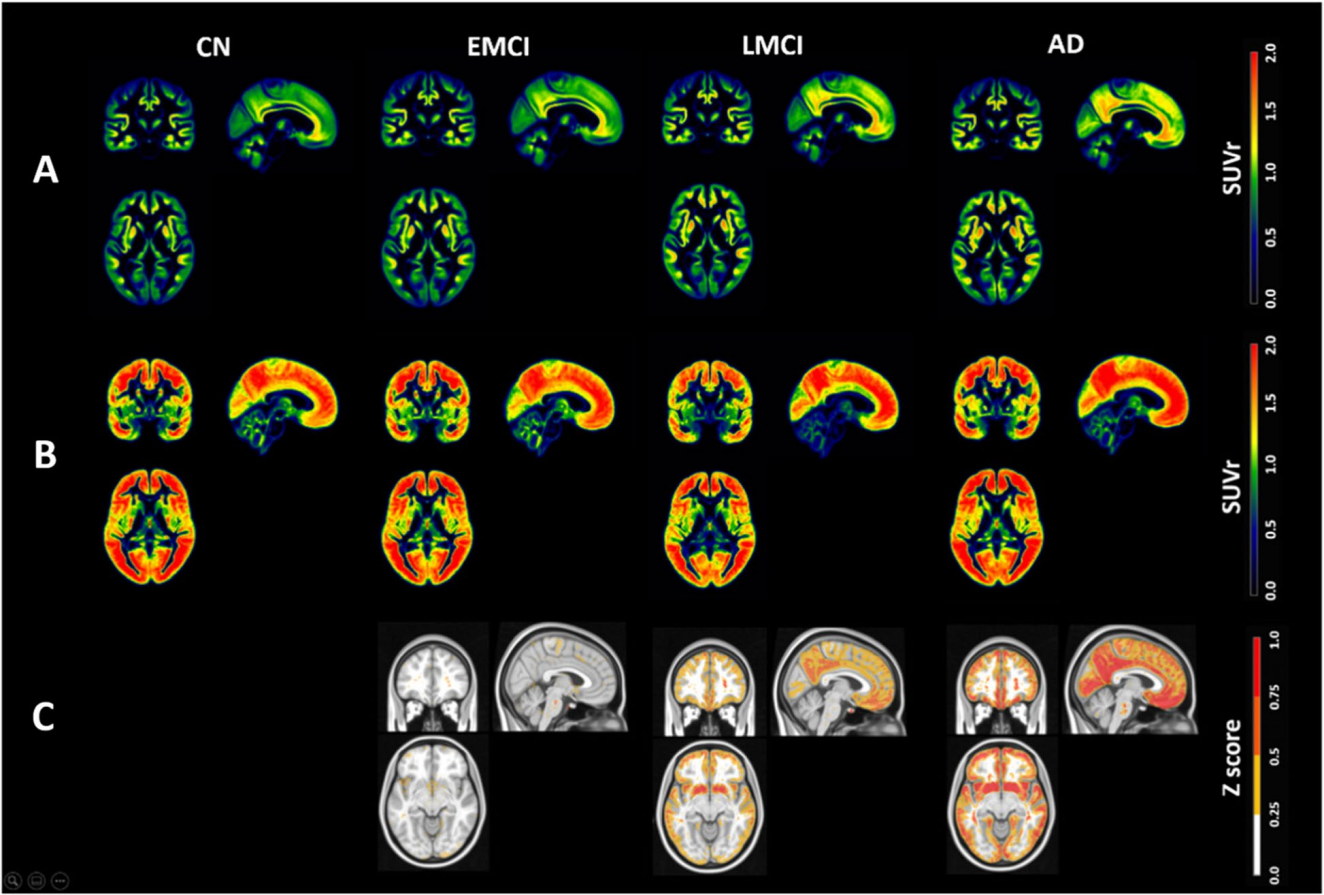
GM masked images showing the mean SUVr (**A**), standard deviation SUVr (**B**), and *Z*-score images overlaid on MNI 152 (**C**) and stratified by clinical group

Quantitative voxel-wise analysis across all scans showed greater uptake in the precuneus, posterior cingulate, isthmus cingulate, and the medial and lateral orbitofrontal cortices in EMCI. Greater uptake was found in lingual, pericalcarine, precentral and post-central cortices in LMCI and AD (see Fig. 2).

*Z*-score images were created of EMCI, LMCI and AD amyloid PET relative to CN. There are large increases in Aβ cluster expansion with clinical progression of disease in the above areas.

### K-means results and mapping clusters to anatomy

**Fig. 3:**
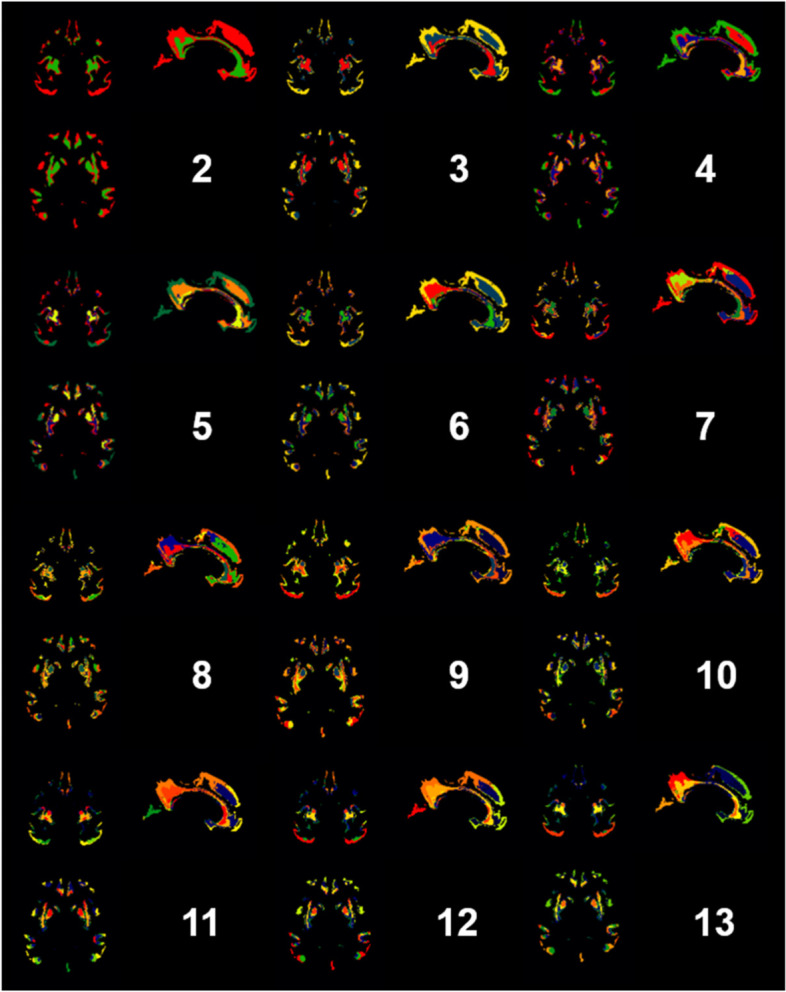
K means voxel-wise clustering results mapped to anatomy. Number corresponds to *k* value. Colours represent different clusters for a given *k* and do not correspond between images when *k* varies

K-means clustering of amyloid PET positive voxels was performed and mapped to anatomy in order to form the classifier (see Fig. 3). Areas that were delineated by clustering included the precuneus, subgenual area, thalamus and anterior and posterior cingulate, prefrontal, occipital and orbitofrontal cortices.

**Fig. 4:**
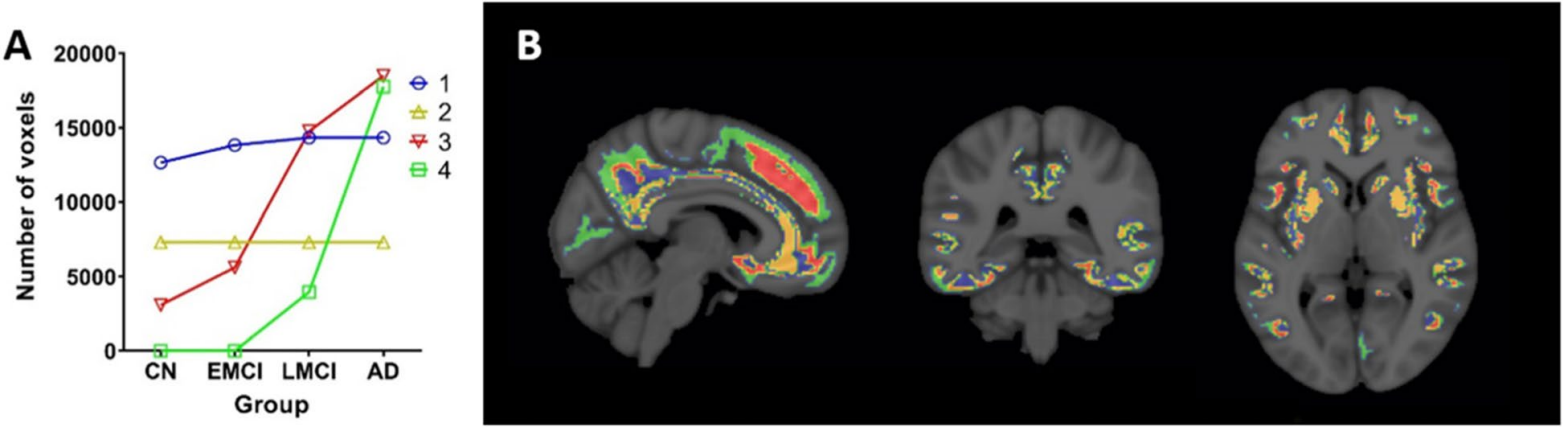
Mean number of voxels of each group (GM masked mean constrained by AD *z*-score mask, thresholded at 1.1) for Aβ clusters 1–4 (**A**). Aβ clusters 1–4 map overlaid on MNI 152 (**B**)

**Fig. 5:**
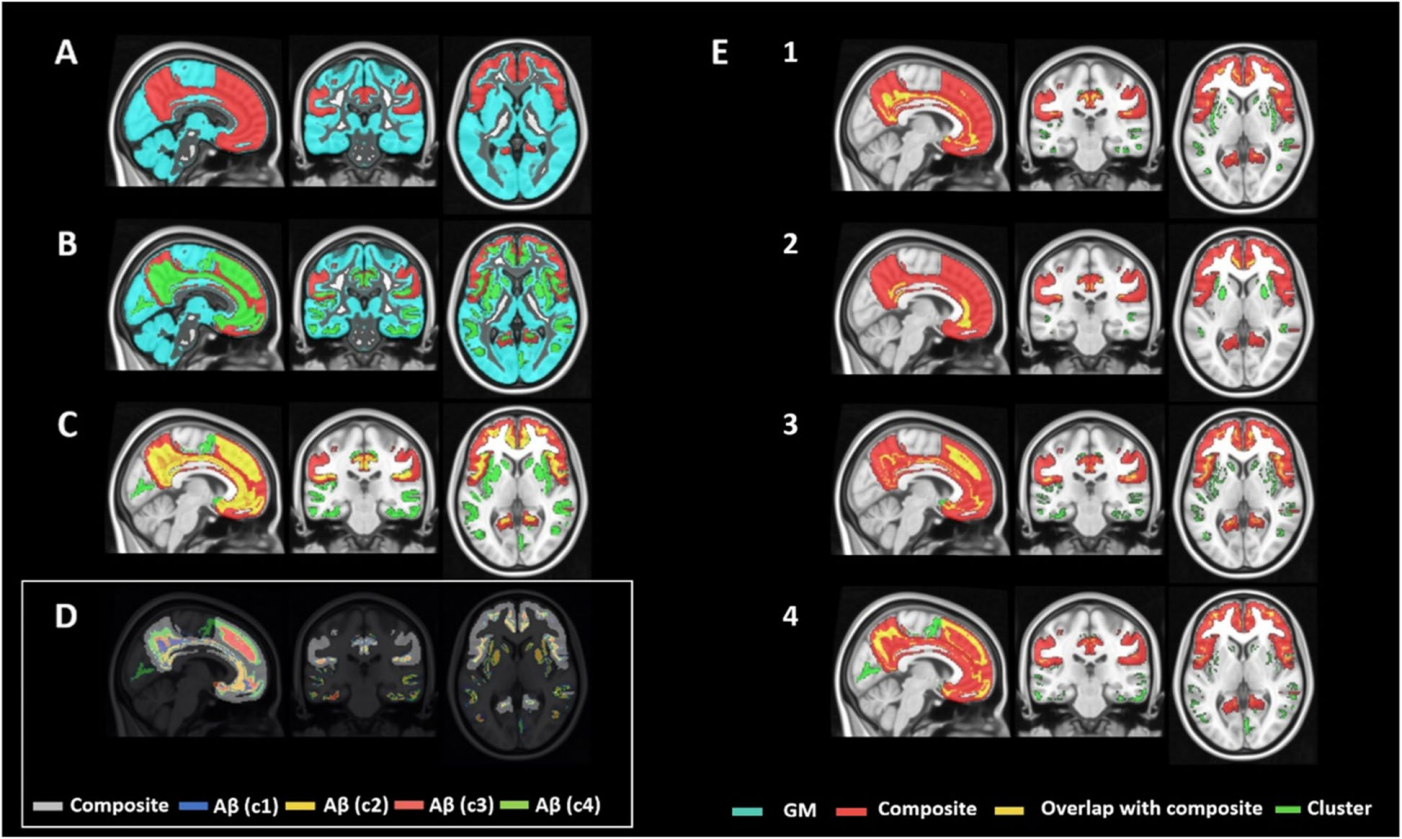
Overlays of composite region of interest, GM and Aβ clusters 1–4 on MNI152. Panel **A** shows composite ROI on GM; panel **B** shows all Aβ clusters overlaid onto composite and GM. Panel **D** shows the relationship together between Aβ clusters 1–4 and composite. Panel **E** shows the Aβ clusters 1–4 individually and the overlap with composite

A parsimonious model (*k* = 4) gave the most discriminative clusters for distinguishing the clinically defined groups of subjects (see Fig. 4), broadly consistent with expectations from cross-sectional pathological assessments *post mortem* [34]. Voxel clusters on silhouette plots for all *k* values tested and their discriminatory capacity are illustrated in Additional file 1: Figure 1 and Additional file 2: Figure 2 (Fig. 4). The relationship of this spatial model with GM and composite regions of interest are shown in Fig. 5. We also found that WM SUVr shows no difference between clinical groups (Additional file 3: Figure 3), reducing the likelihood that spillover from WM contributes to cluster delineation.

### Clinical associations with amyloid PET positive clusters

**Fig. 6:**
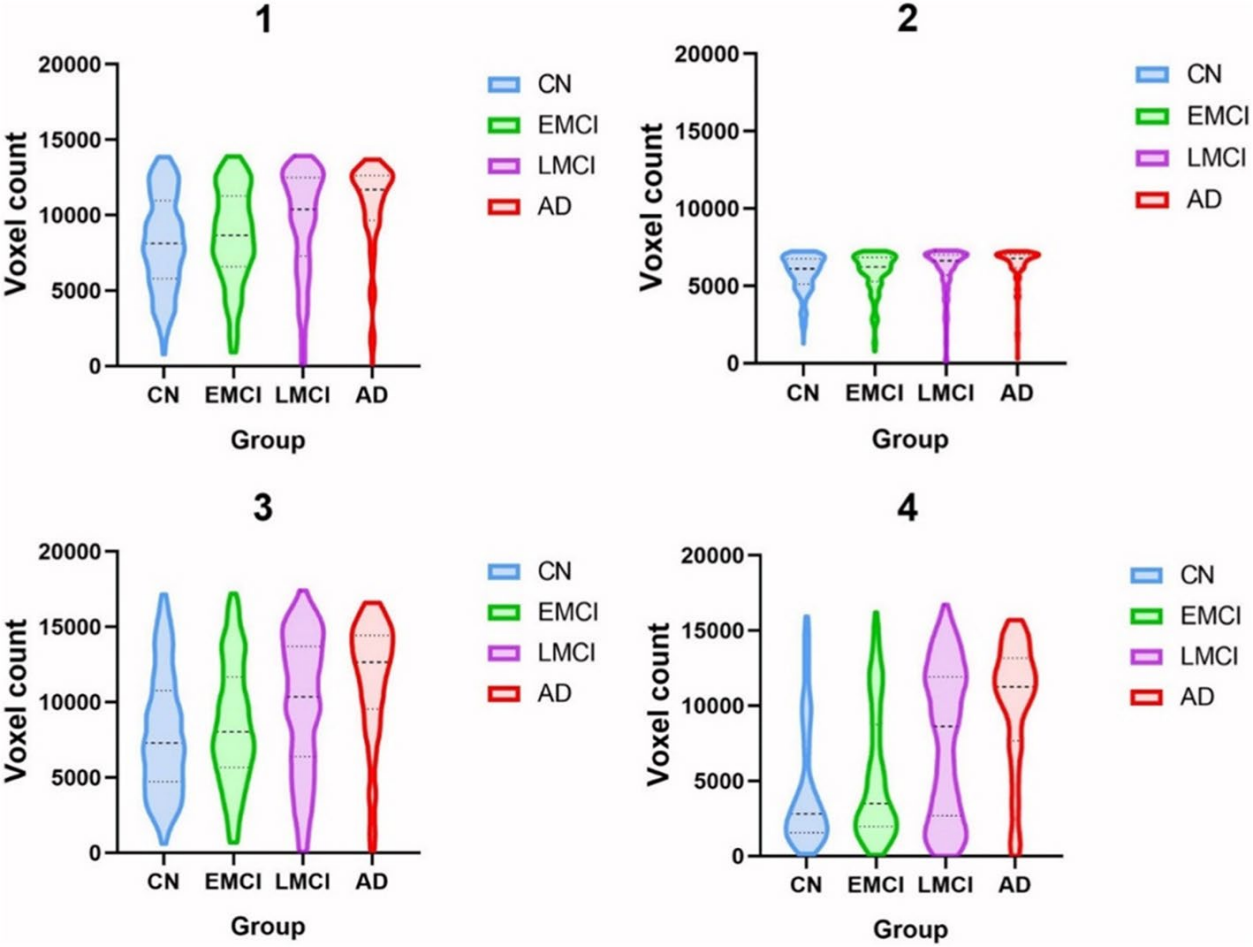
Violin plots showing the distribution of the number of positive amyloid voxels in each individual for Aβ clusters 1–4 stratified by the clinical group. Dashed line indicates the median value, dotted lines divide quartiles, and the width of the probability density for *n* = 758 subjects

Clinical associations of Aβ clusters defined using the *k* = 4 divisions with diagnostic category were explored (labelled as Aβ clusters 1–4). Violin plots were generated using Aβ clusters 1–4 masks applied to each individual in the whole population (Fig. 6) and tabulated (Table 2).

**Table 2:** Median, interquartile range (IQR) and skewness of Aβ-positive voxels in clusters 1–4 across the diagnostic category

	CN Median (IQR)	EMCI Median (IQR)	LMCI Median (IQR)	AD Median (IQR)
1	8126 (5163)	8666 (4682)	10393 (5218)	11685 (2986)
2	6107 (1652)	6229 (1543)	6631 (1292)	6780 (829)
3	7285 (6038)	8028 (5988)	10350 (7309)	12666 (4908)
4	2818 (5370)	3492 (6797)	8633 (9214)	11264 (5498)
	**Skewness g**_**1**_	**Skewness g**_**1**_	**Skewness g**_**1**_	**Skewness g**_**1**_
1	− 0.08	− 0.37	− 0.89	− 1.79
2	− 1.23	− 1.62	− 2.07	− 2.71
3	0.40	0.20	− 0.39	− 1.10
4	1.21	0.83	− 0.08	− 1.01

Cluster 1 showed an increase in the number of Aβ positive voxels with increasing clinical severity. Both CN and EMCI had similar medians with a normally distributed spread across the population. LMCI and AD showed similar distributions with a skew towards higher voxels positive, and a long thin tail towards 0 (AD g_1_ = − 1.79). Cluster 2 had a similar distribution but occupied a smaller area.

Cluster 3 showed a normal distribution in the population of CN and EMCI. LMCI also showed a large spread in Aβ positive voxels across this population with a greater skew towards more positive voxels. The AD group showed a greater skew towards positive voxels (AD g_1_ = − 1.10).

Associations of cluster 4 were markedly different from those of other clusters. CN and EMCI groups within this cluster had a skew towards 0 with a long thin tail extending towards higher voxels positive (CN g_1_ = 1.21). LMCI scans showed a bimodal distribution with 2 peaks, one similar to the CN and EMCI groups at the lower end and a second with a similar distribution to AD at the higher end.

Taken together, these results highlight differences in the distribution of Aβ PET signal clusters 1–4 stratified by the clinical group. The clinically heterogenous MCI populations show a bimodal distribution (either like CN or AD) for cluster 4.

We evaluated the relationships of Aβ PET signal in clusters 1–4 with Mini-Mental State Examination (MMSE) and Clinical Disease Rating (CDR) scores. We found a negative relationship between the signal in all of the Aβ clusters 1–4 and MMSE (Pearson’s *r* values of − 0.24, − 0.14, − 0.25, − 0.36, respectively, *p*<0.001) for 730 individuals where MMSE was documented within 6 months of amyloid PET.

We also found a positive relationship between Aβ clusters 1–4 and CDR score (Pearson’s *r* values of 0.20, 0.10, 0.22, 0.32, respectively, *p* < 0.001–0.004) for 758 individuals where CDR was documented within 3 months of Aβ PET.

### Optimisation of classifier and performance

A quadratic support vector machine (SVM) [33] was chosen as a classifier following optimisation for a combination of multiple features. An optimised linear regression model was chosen as the most accurate classification of single feature composite SUVr and for two features (GM voxels and GM SUVr).

The resultant classifier increased sensitivity by 15%, and the area under the receiver operating curve (AUC) by 9% compared to the “gold standard” composite SUVr. The classifier increased sensitivity by 39%, and the AUC by 20% compared to using the number of amyloid GM voxels positive (thereby adding spatial information) and the unconstrained amyloid GM SUVr (see Tables 3 and 4).

**Table 3:** Classification results comparing sensitivity, specificity, accuracy, AUC-ROC and error rates for different methods

Method	Features	Sensitivity	Specificity	Accuracy	AUC	Error rate
Aβ clusters 1–4, GM voxels and GM SUVR	6	**0.83**	0.81	**0.81**	**0.91**	**0.19**
Aβ clusters 1–4	4	0.74	0.84	0.80	0.87	0.20
Composite SUVR	1	0.67	**0.86**	0.79	0.82	0.21
GM voxels and GM SUVR	2	0.44	0.85	0.69	0.71	0.31

**Table 4:** Percentage change compared to the proposed method - 6 features (Aβ clusters 1–4 and GM voxels and GM SUVr)

Method	Sensitivity	Specificity	AUC
Aβ clusters 1–4	− 8.3	3.0	− 4.0
Composite SUVR	− 15.2	4.9	− 9.1
GM voxels and GM SUVR	− 38.6	4.0	− 19.8

**Fig. 7:**
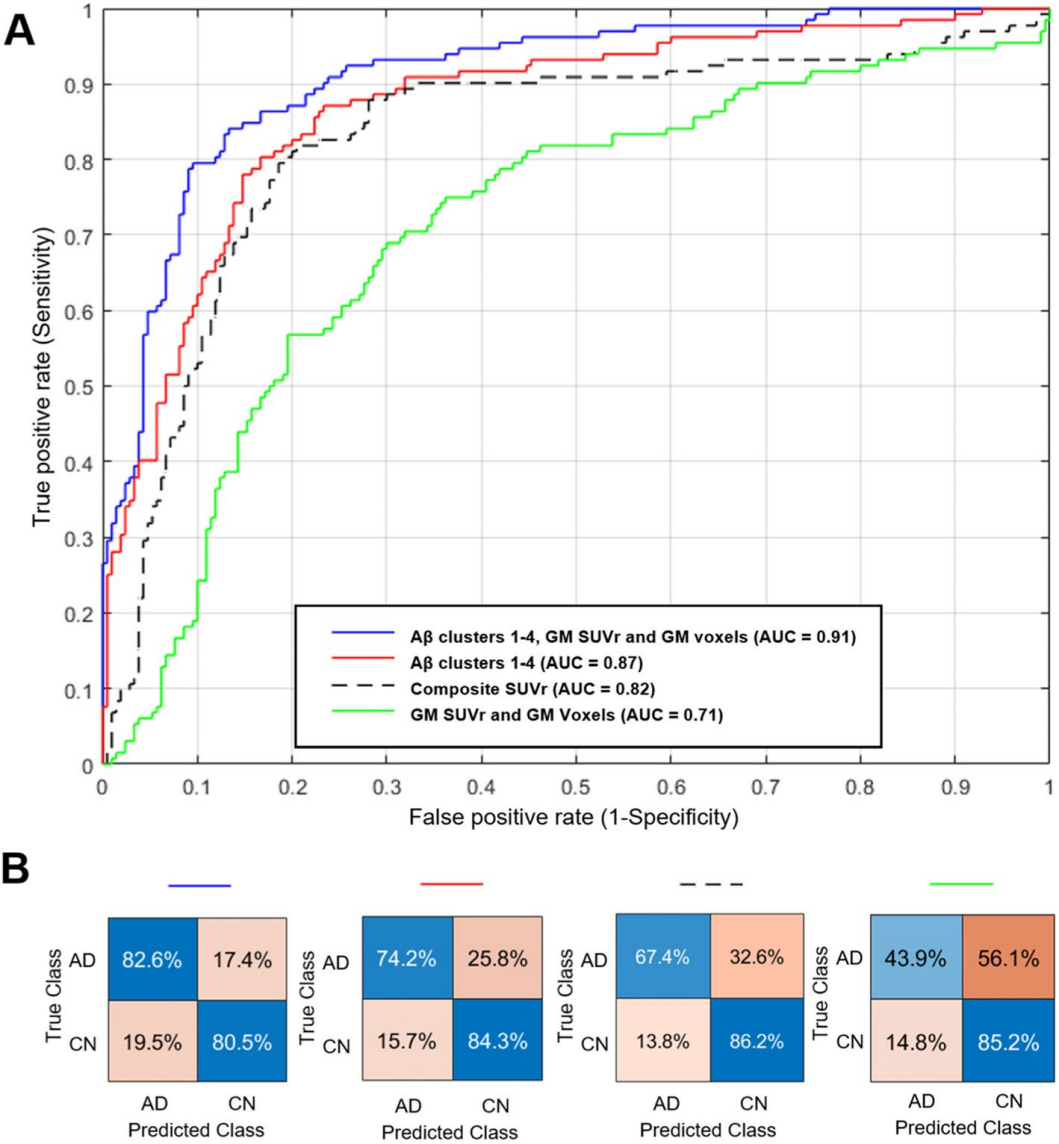
AUC-ROC curve diagram of classification of AD vs CN for Aβ clusters 1–4 compared to and combined with other methods (**A**). The blue ROC curve is created by a combination of 6 features (Aβ clusters 1–4, GM voxels positive and GM mean SUVr) using SVM. The red ROC curve us created by the de novo Aβ clusters 1–4 using SVM. The dashed line ROC curve is a composite SUVr, and the green line is 2 features of GM voxels positive and GM mean SUVr using linear regression. Confusion matrices (**B**) with percentages for the corresponding ROC curves are on the right panel. These show the percentage of AD and CN individuals correctly predicted (blue) and incorrectly predicted (orange) for each method

The optimum combination of 6 features (Aβ clusters 1–4 and GM voxels and GM SUVR) boosted the accuracy of the classifier to classify AD to 81% using amyloid PET alone, with the highest sensitivity (0.83) and lowest error rate (AUC 0.91) relative to all other methods (see Fig. 7).

## Discussion

We have generated a data-driven algorithm that provides an automated classification of amyloid PET scans from people across the AD continuum that outperforms existing spatial measures tested here to boost the independent diagnostic power of amyloid PET. This automated classifier potentially could enhance the efficiency and accuracy with which amyloid PET scans are interpreted by radiologists and clinicians in the context of cognitive decline.

Our approach, derived from a data-driven model of amyloid PET positive clusters was optimised diagnostically and used to generate a classification system. In doing so, we improved the spatial sensitivity of amyloid PET to detect AD to 0.83, with a high AUC of 0.91 using an optimised support vector machine. Use of a *k*-fold cross-validation prevented the overfitting associated with hold out validation and is powerful as trained over a large number of amyloid scans. The classifier was trained using data from across multiple sites to capture variances typical for multi-centre and multi-scanner datasets across the AD continuum. The algorithm will be available online (github.com/brainregion) for evaluation as a tool for automated clinical decision support in research and as an adjunct to practice.

In developing our classifier, we had the opportunity to explore disease staging by mapping the trajectory of in vivo Aβ cluster expansion in AD using data-driven clustering of amyloid PET. *Post mortem* evidence shows that Aβ plaque deposition spreads neocortically, moves to allocortical areas, then diencephalic nuclei, and finally cerebellum, [35] with associated patterns of neurofibrillary tau tangle deposition [4, 35]. Newer in vivo PET approaches to assess fibrillar Aβ-beta have been useful for assessing in vivo pathology; however, staging systems have either attempted to replicate classification systems used for *post mortem* neuropathology or are limited by the constraints of atlas-based approaches [36, 37]. Our approach differed from traditional approaches and uniquely assessed voxels positive within novel Aβ clusters, as well as having an AD training set that is specific to Aβ pathology in AD. The Aβ clusters of a constrained area yielded higher accuracy, compared to a larger composite ROI [26, 27, 38] (the primary outcome measure in many studies) or whole GM. Enlargement, rather than absolute intensity, is able to evaluate unique properties of Aβ accumulation through cluster growth.

There are several advantages of using an “explainable” model rather than “black-box” approaches of deep-learning methods used previously for similar classification questions [39–41]. Foremost, amongst these are the ease of interpretability of the spatial features derived from clustering and its relative simplicity of implementation. It also facilitates the assessment of the relationships of measures to well-described pathological and clinical stages of the disease. Other approaches combine different amyloid tracers [40], are mixed with other PET targets to achieve higher accuracy [41], or focus on visually equivocal cases [39] and use the standard cortical SUVr mask, which our spatial model outperforms.

By exploring their clinical associations we found that a parsimonious model distinguishing 4 clusters was able to distinguish early from late disease and show differences in the distribution of positive Aβ voxels between disease groups. Usual clinical “visual” reads do not support this level of discrimination, which can be important for research applications, as well as having clinical relevance. This has relevance in that there may also be value in using different parts of the brain to track the progression of the disease—for example, Aβ cluster 4 to evaluate track LMCI progression given the bimodal distribution and understanding the regional vulnerability or inflexion point at which MCI converts to AD. Previous literature has evaluated Aβ in MCI but on smaller numbers [42]. This result may also reflect the diagnostic uncertainty of an MCI diagnosis, of which other causes need to be rule out, and the utility of Aβ PET to change management in an MCI population [13].

Future work involves testing the utility of using different cluster regions to assess change at different clinical severity timepoints in response to potential disease-modifying therapeutics for AD in clinical trials.

### Limitations

One limitation of this study is that it derives data inferring progression based on a cross-sectional research derived dataset with Aβ PET signal accompanying MRI. However, over the long prodromal period of sporadic late-onset AD, cross-sectional analyses have been shown to predict longitudinal change well [32, 43]. Future work will involve replication in other cohorts to understand the extent to which results from these research-domain data can be generalised to usual clinical imaging. Other limitations include that we uniquely looked at the expansion of clusters over a cut-point with data-driven clusters and not the change in intensity at a voxel-wise basis. An advantage of our approach is that traditional SUVr analyses are not able to pick up expansions in volume of amyloid clusters. Most larger cohort studies in AD lack histopathologically confirmed ground truth diagnoses. This is a limitation to the accuracy of the classifier; however, the large number of scans trained across multiple sites may help reduce the impact of site-specific inaccuracies. The radiotracer for scans employed in our study is [^18^F]Florbetapir, which is one of the most widely used amyloid tracers. The binding site appears to be similar to that of other Aβ tracers [44]. We do not expect quantitative differences with other Aβ tracers [31] but this would need further testing.

## Conclusions

Accurate diagnosis of AD in clinic is difficult given the variety of neuropsychiatric symptoms, the varying trajectories of these, and confounding non-AD effects on cognition. We have developed a classifier that promises to boost the diagnostic power of amyloid-β PET using a data-driven model of amyloid cluster progression with AD. This algorithm, which is based on the progression of regional brain vulnerabilities to AD, provides automated diagnostic decision support that outperforms classifiers proposed previously for amyloid-β PET based on spatial constraints. The algorithm and its de novo cluster masks may be helpful for clinicians and radiologists investigating cognition in clinic.

## Supplementary Information

**Additional file 1: Figure 1**. Number of voxels of each group (GM masked mean*ADzmap mask, thresholded at 1.1) within each k division.

**Additional file 2: Figure 2**. Silhouette plots for 57929 voxels across 758 subjects showing how close each voxel is in one cluster to voxels in neighbouring clusters.

**Additional file 3: Figure 3**. WM SUVr shows no difference between clinical groups.

## Data Availability

The datasets analysed during the current study are available from the Alzheimer’s Disease Neuroimaging Initiative (ADNI) http://adni.loni.usc.edu/.
