# Supplementary figures and images for: Boosting the diagnostic power of amyloid-β PET using a data-driven spatially informed classifier for decision support
Source: Alzheimers Res Ther. 2021 Nov 10;13:185. doi: 10.1186/s13195-021-00910-8 (PMC8582159; doi:10.1186/s13195-021-00910-8)

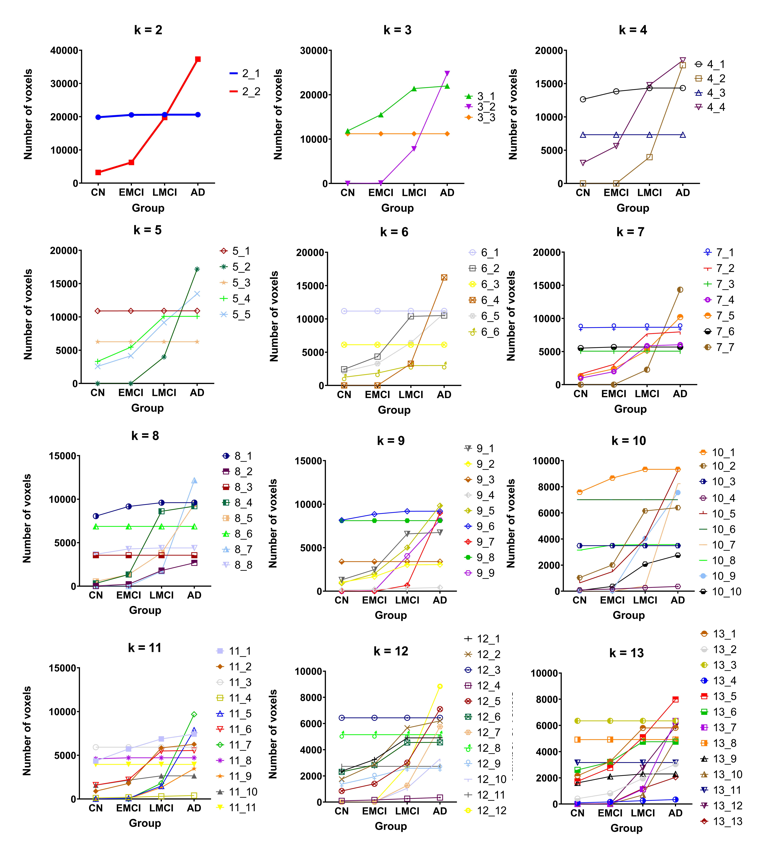

Supplement: Supplementary file 1 — Additional file 1: Figure 1. Number of voxels of each group (GM masked mean*ADzmap mask, thresholded at 1.1) within each k division. [file 13195_2021_910_MOESM1_ESM.docx]

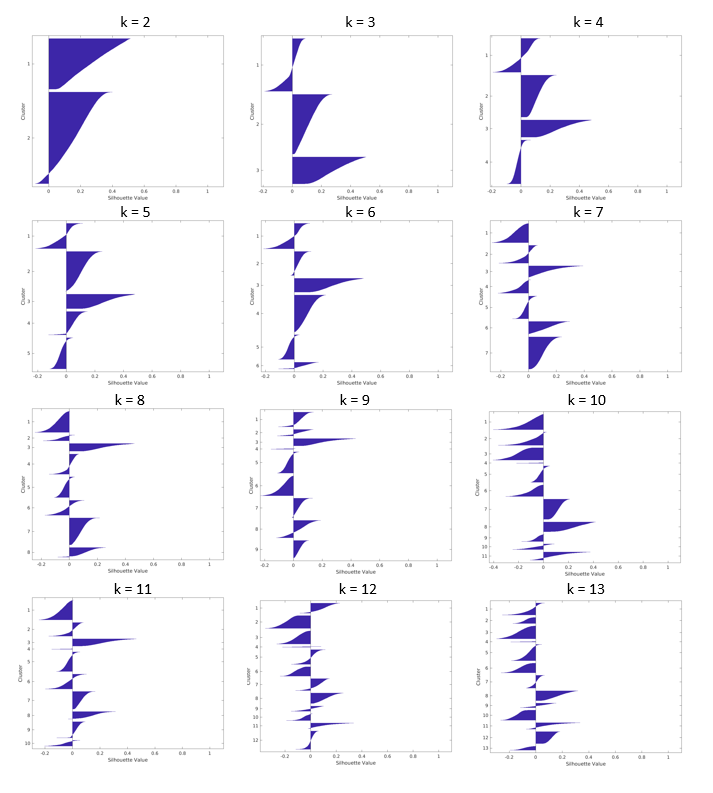

Supplement: Supplementary file 2 — Additional file 2: Figure 2. Silhouette plots for 57929 voxels across 758 subjects showing how close each voxel is in one cluster to voxels in neighbouring clusters. [file 13195_2021_910_MOESM2_ESM.docx]

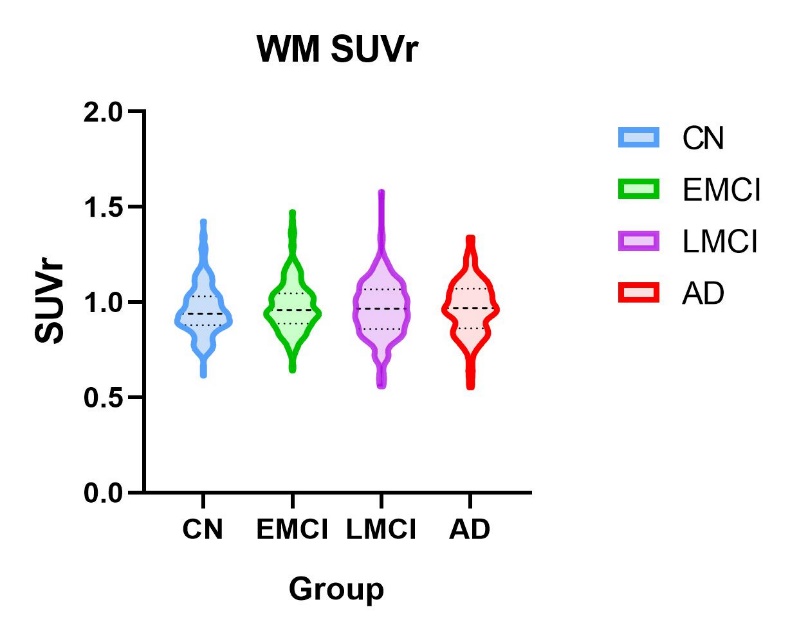

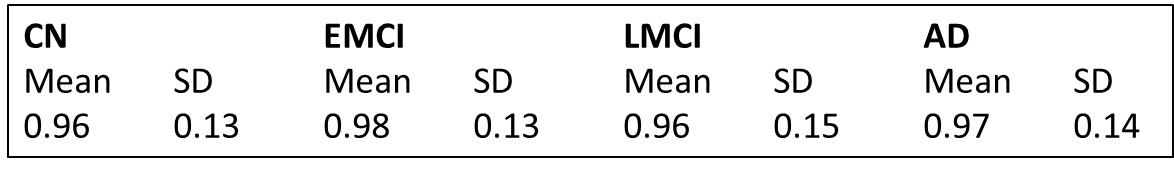

Supplement: Supplementary file 3 — Additional file 3: Figure 3. WM SUVr shows no difference between clinical groups. [file 13195_2021_910_MOESM3_ESM.docx]
